# Gene editing in birds takes flight

**DOI:** 10.1007/s00335-017-9701-z

**Published:** 2017-06-13

**Authors:** Mark E. Woodcock, Alewo Idoko-Akoh, Michael J. McGrew

**Affiliations:** The Roslin Institute and Royal Dick School of Veterinary Studies, University of Edinburgh, Easter Bush Campus, Midlothian, EH25 9RG UK

## Abstract

The application of gene editing (GE) technology to create precise changes to the genome of bird species will provide new and exciting opportunities for the biomedical, agricultural and biotechnology industries, as well as providing new approaches for producing research models. Recent advances in modifying both the somatic and germ cell lineages in chicken indicate that this species, and conceivably soon other avian species, has joined a growing number of model organisms in the gene editing revolution.

## Bird germline transgenesis

The chicken has been an exceedingly useful model for the study of early vertebrate development and patterning (Stern 2005). As the new genome editing technologies are applied to bird species, it is certain that these research efforts will provide new insights into avian diseases, reproduction, growth and nutrition, and beyond. The advent of gene editing of the avian genome follows on from over 30 years of transgenic research in chickens. Transgenesis in birds has always lagged behind the advances made in other vertebrate species because of the inaccessibility and complex yolky structure of the avian zygote (Romanoff and Romanoff 1949). Many of the early technical advances in avian transgenesis used microinjection of retroviruses, culminating in lentiviral vectors, to achieve efficient germline modification without subsequent vector silencing (Salter et al. 1987; Bosselman et al. 1989; Page et al. 1991; McGrew et al. 2004; Lee et al. 2007). More recently, direct electroporation and lipofection of DNA transposons or site-specific DNA recombinases into early developmental stage embryos have been used for the non-targeted integration of transgenic constructs in both the germ lineage and somatic tissues of the chicken (Kong et al. 2008; Takahashi et al. 2008; Serralbo et al. 2013; Tyack et al. 2013; Jordan et al. 2014). The use of these techniques in transgenic studies has already been extensively reviewed (McGrew 2013; Nishijima and Iijima 2013; Collarini et al. 2014). These previous studies have increased our knowledge of immune function (Thompson et al. 1987; Sayegh et al. 1999) and embryonic development (Sato et al. 2007; Macdonald et al. 2012; Glover et al. 2013), and have led to the development of new disease models (Dodgson and Romanov 2004; Wick et al. 2006; Williams and Bohnsack 2015).

## Gene editing tools

The field of functional genomics was transformed with the arrival of zinc-finger nucleases, allowing the efficient targeted integration of transgenes, or the introduction of targeted mutations to the genome (Bibikova et al. 2002; Fan et al. 2011). Now, breakthroughs with TALEN and CRISPR/Cas9 technology permit genome editing with rapid construction of targeting plasmids and at lower costs. Direct injection of editors into zygotes also replaces the need for the culturing of embryonic stem cells as intermediaries in the process of producing genetically altered offspring.

**Fig. 1:**
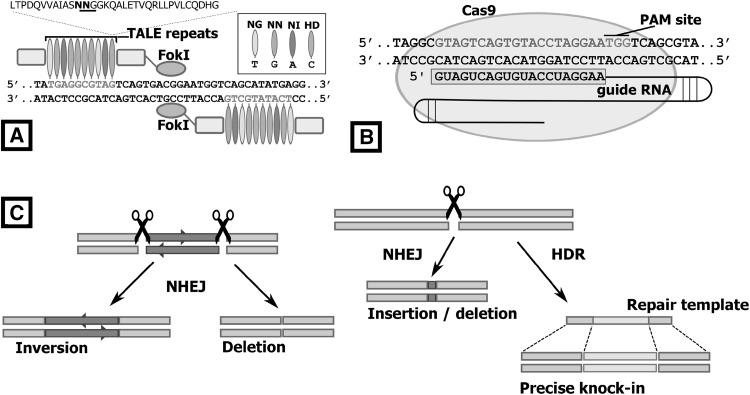
TALENs and CRISPR/Cas9 target DNA and generate genomic edits through the NHEJ and HDR repair pathways. **a** TALE proteins consists of repeated modules, fused to non-specific FokI cleavage domains that generate double-stranded DNA breaks upon dimerisation. Each repeated unit differs at amino acids 12 and 13, and the dipeptide combination at this position determines the nucleotide-binding specificity. **b** The CRISPR/Cas9 complex includes a 20-nucleotide guide RNA (gRNA) that guides Cas9 to the target DNA. Cas9 nuclease activation requires a PAM (NGG) sequence to lie immediately downstream of the target DNA. The bound RNA complex activates double-stranded cleavage through two domains on the Cas9 nuclease, at a position close to the PAM site. **c** Breaks in the DNA are then repaired by the NHEJ or HDR pathway

TALEs (Transcription activator-like effectors) are naturally occurring proteins from the plant pathogenic bacteria genus Xanthomonas, and contain DNA-binding domains composed of a series of repeat units 33–35 amino acids long, with each unit recognising a single base-pair, depending on two highly variable residues in the middle of each unit (Boch et al. 2009; Moscou and Bogdanove 2009). By fusing the DNA-cleavage domain of FokI onto a TALE, TALE nucleases (TALENs) were produced and shown to be applicable to gene editing outside their native plant-host system for generating genetic changes by both non-homologous end joining (NHEJ) and homology-directed repair (HDR) (Fig. 1) (Miller et al. 2011; Wood et al. 2011). Injection of mammalian zygotes with TALEN mRNA followed by transfer to surrogate host animals has been successful in producing genome-edited animals, including rat and mice (Wang et al. 2013; Ponce De León et al. 2014), as well as in mammalian livestock species such as pigs and cattle (Carlson et al. 2012; Lillico et al. 2013).

The CRISPR/Cas9 system, based on the CRISPR-Cas adaptive immune system found in a number of bacterial and archaeal species (Jinek et al. 2012), uses small non-coding RNAs to guide the Cas9 nuclease to a target site in the eukaryotic genome, where it then cleaves the double-stranded DNA target. The guide RNA usually contains a 20 nucleotide sequence complementary to the target site, with the target site further restricted by the required presence of an adjacent 3-nucleotide sequence termed the protospacer adjacent motif (PAM). The ease of synthesising and cloning custom guide RNAs for Cas9 recognition is an improvement upon the sequential cloning protocols needed to produce custom DNA-binding domains for TALENs (Cermak et al. 2011). Although CRISPR/Cas9 is a quick and flexible tool for the recognition and cutting of specific genomic sites, it is met with similar problems encountered by TALENs when applied to avian germline transgenesis. Transferring this technology to avian embryos is confounded by the difficulties in accessing the early avian zygote in the hen, and subsequently supporting the developing embryo post-injection/transfection (Perry 1988; Sherman et al. 1998). For this reason, most genome editing studies reported so far for birds have described the application of CRISPR/Cas9 to avian somatic cells and tissues.

## Gene editing of avian somatic tissue and cells

The somatic chicken B cell line, DT40, has proved invaluable for providing insights into the function of adaptive immunity, and has been key for investigating the genetic mechanisms involved in repairing double-stranded breaks in the genome by both the NHEJ and HDR pathways (Takata et al. 1998; Brown et al. 2003). These cells are unusual as they exhibit higher frequencies of targeted versus random integration of transgenes into the genome, in comparison to other somatic vertebrate cell lines (Buerstedde and Takeda 1991). For this reason, these cells have been particularly useful for loss-of-function and protein-tagging *in vitro* studies (Chavali and Gergely 2015; Kobayashi et al. 2015; Daly et al. 2016).

DT40 cells and chick embryonic fibroblasts (such as the DF-1 cell line) have also been instrumental in developing the application of CRISPR/Cas9 to the chicken. Initial transfection studies demonstrated the efficient creation of directed mutations in numerous chicken loci across both micro- and macrochromosomes, and the removal of large continuous sections (>75 kb) of the genome in these cell lines (Bai et al. 2016; Dad Abu-Bonsrah et al. 2016). By employing Rad52, an HDR-enhancing element, researchers demonstrated a greatly improved efficiency of CRISPR/Cas9 editing in DF-1 cells (Wang et al. 2017). Furthermore, researchers have used conserved avian promoter modules to drive Cas9 protein expression in the somatic tissue of other bird species, thus introducing NHEJ edits to the MLPH locus of a quail myoblast cell line (Ahn et al. 2016). The use of CRISPR/Cas9 in genetic studies has also been demonstrated in avian cell lines; frameshift deletions in the Grb2 locus were used to analyse B cell receptor signalling in DT40 cells, and a CRISPR/Cas9 deletion analysis provided insight into interactions between the surface receptor CD22 and plasma membrane transport proteins in DF-1 cells (Chen et al. 2016).

Additionally, CRISPR/Cas9 has been useful for the in ovo study of gene function in developing somatic tissues. It is relatively easy to introduce DNA vectors into chicken embryonic cells by direct electroporation of developing tissues of the chicken embryo for the study of spatio-temporal gene functions, for example, the somites, the primitive streak and the cranial neural crest (Marcelle et al. 1995; Dubrulle et al. 2001; Bronner-Fraser and García-Castro 2008). Electroporation of CRISPR/Cas9 vectors into embryos lead to lineage-specific loss-of-function, and chimaeric chicken embryos produced in this manner were used to study loss-of-function of genetic targets in the developing neural tube and somites (Véron et al. 2015). Strikingly, this remains the only published report for the in ovo electroporation of CRISPR/Cas9 vectors into chicken embryos.

So far, the in ovo editing of chicken embryos has not produced genetic modifications that have been transmitted through the germ cell lineage to offspring. This may be due to the distinct developmental ontogeny of the avian germ cell lineage. Yet, by directly targeting chicken primordial germ cells (PGCs), it is possible to introduce specific edits into the chicken genome, and to use these edited germ cells to produce gene-edited (GE) chickens. In the past, the genetic manipulation of germ cells has been hampered by impracticalities of targeting these cells in vivo and the inability to propagate PGCs in vitro. However, recent advances in culturing PGCs have enabled the efficient generation of GE germ cells which can subsequently be used to generate GE chicken.

## Primordial germ cells for genome editing

**Fig. 2:**
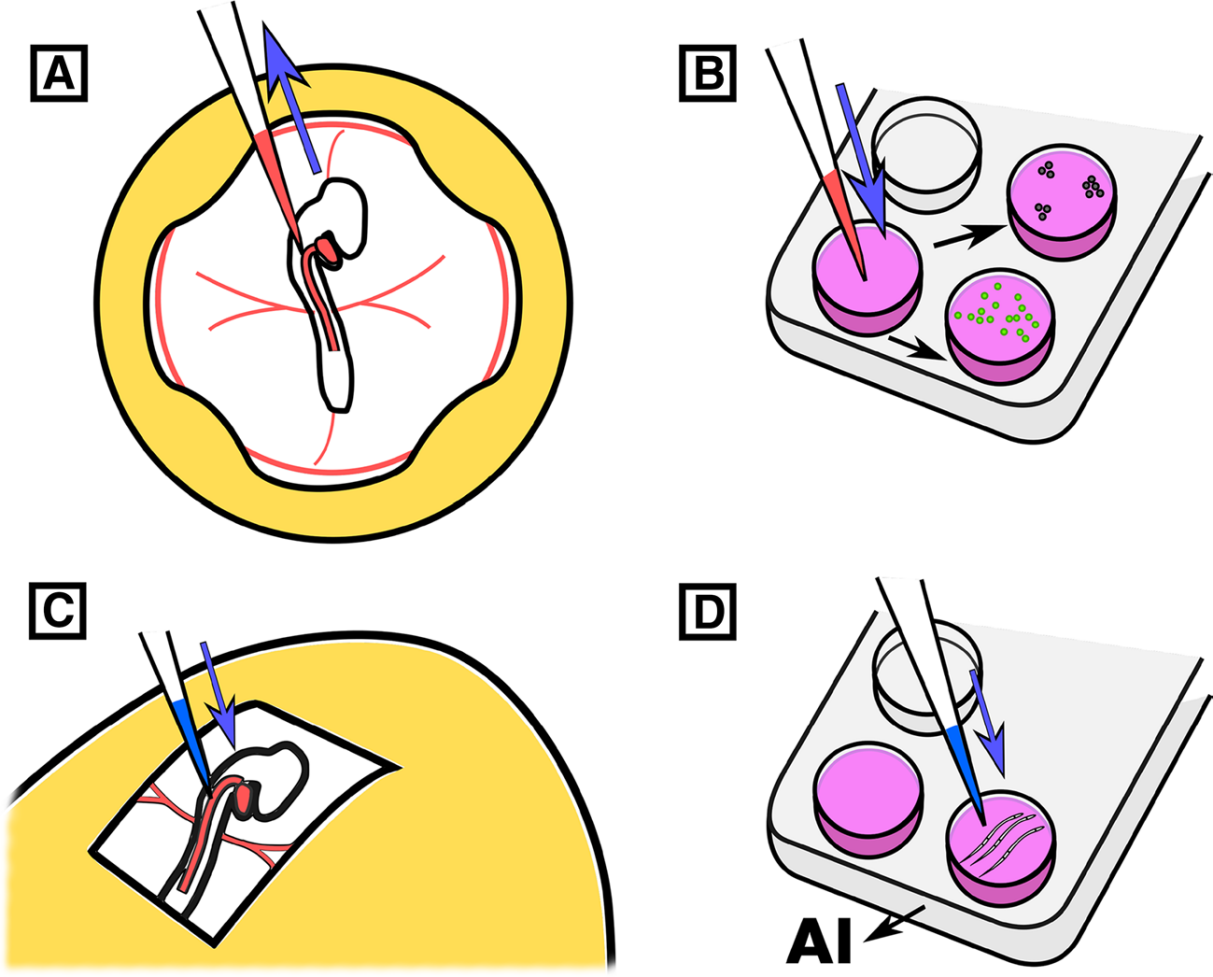
Targeting the avian germ lineage. **a** Derivation and transfection of avian PGC cultures. Blood is collected from the embryo once PGCs have begun circulating in the vasculature. Derived PGC cultures can then be transfected via lipofection or electroporation. **b** PGCs are purified with a selectable marker, or by culturing and sequencing clonal populations. Use of a semi-defined culture media increases proliferation in vitro, with potential increases to gene targeting efficiency. **c** Injection of surrogate embryos with transfected PGCs. Transfection reagents can also be injected at this stage to edit germ cells in ovo. **d** Semen transfection with CRISPR/Cas9 constructs is subsequently used for artificial insemination (AI)

Current evidence supports the hypothesis that avian PGC specification occurs through maternal factors deposited as germplasm in the developing oocyte (Petitte et al. 1997). In the laid chicken egg, the embryonic blastoderm consists of approximately 60,000 cells, containing 40 PGCs clustered in the centre of the disc, as evidenced by the expression of PGC-specific nucleic acid-binding proteins *DDX4* and *DAZL* (Tsunekawa et al. 2000; Lee et al. 2016). In the first 12 h of incubation, PGCs translocate to the forming hypoblast, and then migrate anteriorly as the primitive streak elongates from the posterior border of the blastoderm. The PGCs come to reside within the germinal crescent, the mesoderm located anterior to the head fold, where they remain until the extraembryonic blood islands form in that region. At 48–60 h of incubation, a population of approximately 100–200 PGCs enters the circulatory system and exit into the lateral plate mesoderm to finally migrate to the genital ridge of the nascent gonads (Ginsburg and Eyal-Giladi 1986; Nakamura et al. 2007). PGCs are accessible at many of these stages of development, either through dissection of the embryo or aspiration of embryonic blood. Genetic manipulation of PGCs during short-term culture, using standard transfection reagents, can be achieved and used for transgenesis (Hong et al. 1998). More recently, it was shown that circulating PGCs can be targeted directly via intravenous injection of transfection reagents to produce transgenic offspring (Tyack et al. 2013) (Fig. 2). While in this case a transposon vector was used for the genomic insertion of a GFP reporter, it seems plausible that this technique could be adapted for use of TALEN or CRISPR/Cas9 vectors, and provide a platform for gene editing in other bird species. Germline transmission using these techniques was infrequent so it is not clear if gene editing of avian PGCs in ovo will be possible. However, the chicken is one of the few vertebrate species for which the long-term in vitro propagation of primordial germ cells is possible, so performing gene editing of cultured PGCs is becoming a standard practice.

The in vitro culture of PGCs is possible using a complex medium containing chicken and bovine serum, conditioned media, feeder cells and growth factors such as FGF2 (van de Lavoir et al. 2006; Choi et al. 2010, MacDonald et al. 2010). Recently, it has been shown that a feeder replacement medium containing growth factors to activate the FGF, insulin and TGF-β signalling pathways could be used to propagate PGCs (Whyte et al. 2015). Furthermore in this report, use of ovotransferrin as a replacement for the iron-carrying proteins present in avian serum permitted feeder-free and serum-free propagation of PGCs, with cells maintaining a high rate of proliferation. A rapid rate of in vitro cell division also aids genome editing experiments, as it increases the potential number of HDR targeting events (Fig. 2).

To produce GE chickens from PGCs edited in vitro, the exogenous edited germ cells are injected intravenously into surrogate host embryos, at a stage when their endogenous PGCs are migrating to the genital ridge. The edited ‘donor’ PGCs must remain viable and outcompete the endogenous PGCs if they are to colonise the forming gonad and transmit the edited chromosome(s) through the germline. To provide donor PGCs with an advantage, the number of endogenous PGCs can be reduced by chemical or genetic ablation (Smith et al. 2015). Exposing the blastoderm of surrogate embryos to emulsified busulfan has been shown to increase germline transmission of donor PGCs to over 90%, though this rate drops significantly if PGCs have been cultured or cryopreserved (Nakamura et al. 2008; Naito et al. 2015). In other animal species, transgenes have been used to successfully target the germ lineage for ablation (Xu and Chisholm 2016). This genetic strategy permits the direct ablation of germ cells when a transgene is expressed or translated specifically in the germ lineage. In salmon, CRISPR/Cas9 was used to knock out the gene *dead end*, a germ cell survival factor, generating fish which lacked germ cells (Wargelius et al. 2016). Thus, ablation of endogenous germ cells by means of chemicals, transgenes or deletion of gene products critical to PGC survival, could improve the efficiency of the production of GE birds from exogenous edited PGCs.

## GE chickens

To date, there has been less than a handful of published reports on the use of TALENs and CRISPR/Cas9 vectors to produce GE chickens, the majority of which make use of cultured PGCs to introduce genetic modifications into the chicken genome. Park and colleagues used TALENs to generate indel mutations at the beginning of the ovalbumin (OVA) gene coding sequence (Park et al. 2014). Somatic DF-1 cells were used to optimise the TALEN vectors, and a transient GFP reporter plasmid was co-transfected to permit fluorescence-sorting of transfected cells. Interestingly, although the mutation rate between DF-1 cells and PGCs was similar in this study, DF-1 cells showed a greater variety of indels, whereas only deletions were observed in the PGCs. 41% of progeny were derived from the donor PGCs, and 8% of the offspring contained mostly nonsense frameshift mutations.

Oishi and co-workers also targeted the OVA locus, and a second locus, ovomucoid (OVM), another albumen protein, using CRISPR/Cas9 vectors, and used transient antibiotic selection to purify the transfected PGCs (Oishi et al. 2016). Semen from founders containing these donor PGCs and the subsequent offspring showed a number of deletions (1–31 bp) at the targeted sites of the OVM locus. Again, no insertions were observed, nor were any edits detected at off-target locations. The researchers also obtained high transmission rates, with 73% of progeny deriving from the donor PGCs and 53% of these contained deletions in the OVM locus.

Dimitrov and colleagues were the first to report HDR editing in chicken by CRISPR/Cas9, using a chicken line which had previously been targeted at the JH segment of the IgH locus using classical homologous recombination (Dimitrov et al. 2016). A site upstream of the VH segment of the IgH locus was targeted in PGCs from the JH-knockout line, and an additional loxP site and antibiotic selection marker were inserted using HDR. Germline transmission rates varied between PGC lines, as well as between injected founder birds containing the same donor germ cells. Most founder birds transmitted the edited allele at a frequency between 0 and 16%, although two founders, injected with the same clonal PGC line, transmitted at higher rates, 36 and 96%.

Recently, TALENs were used to target the *DDX4* locus in chicken PGCs (Taylor et al. 2017). *DDX4* is located on the chicken Z sex chromosome and the mRNA is only expressed in the germ cell lineage. Efficient HDR (8%) of a GFP-puromycin reporter construct was achieved in cultured PGCs, and the targeted cells showed no expression of *DDX4*. Targeted female progeny (ZW) were hemizygous for *DDX4*, and were also found to be sterile. In these hens, the germ cells were present at early developmental stages and later lost during meiosis, indicating the requirement of *DDX4* for oocyte differentiation.

An interesting alternative to the use of PGCs is the direct transfection of spermatozoa using CRISPR/Cas9 vectors (Fig. 2). The CRISPR/Cas9 vectors are thought to target the male or female genome either during sperm decondensation or during syngamy in the post-fertilisation oocyte. DF-1 cells were used to optimise targeting to 200-bp regions in either a GFP transgene or the sex determination factor, DMRT1. Deletion in these coding sequence regions were found in 15% of GFP-targeted cells and 9% of DMRT1-targeted cells (Cooper et al. 2016). However, insemination of transfected sperm from GFP and wild-type roosters resulted in progeny with single base substitutions and short insertion edits (1–5 bp). Inexplicably, indels were located approximately 25 bp 5′ to the predicted CRISPR/Cas9 cleavage sites. Transmission rates of indels from a GFP transgene to chicks produced from the transfected sperm were 14%, while edits to the DMRT1 locus were found only in embryos targeted at a single location in the coding sequence, and co-transfected with a 100-bp HDR oligo (three embryos, 4% of total), though no actual HDR edits were detected. Although the transmission rates were relatively low, transfecting sperm with CRISPR/Cas9 vectors to the zygote removes the need to transfect cultured PGCs, greatly reducing the time taken to produce genetically modified birds. It may also be relatively simple to adapt this technique to introduce genetic modifications into other poultry and bird species, although the precise position of the resulting indels appears unpredictable.

Genome editing in chicken is an emerging field and examples of gene editing in bird species other than chicken are currently lacking. This is likely to be addressed once there is improved knowledge on the culture requirements for non-chicken avian PGCs, and further demonstrations of the efficiency of gene editing in somatic cells or tissues will surely follow.

## Applications for gene editing in birds

### Bioreactors

One of the major aims of early research to genetically modify the chicken was to develop the use of the chicken egg as a bioreactor for producing recombinant proteins (reviewed in Lillico et al. 2005). The advantage of the chicken egg over other mammalian bioreactor systems is that the evolutionary distance between birds and mammals makes it possible to produce many chemo-active mammalian recombinant proteins in birds that will not be recognised by avian cells and organ systems. Additionally, the egg is a sterile self-contained environment with low protease activity and chicken flocks offer favourable scale-up time and relatively low animal costs. The egg bioreactor platform is currently used for the production of human flu vaccine, which means existing regulatory procedures can be adapted for the new bio-products.

**Fig. 3:**
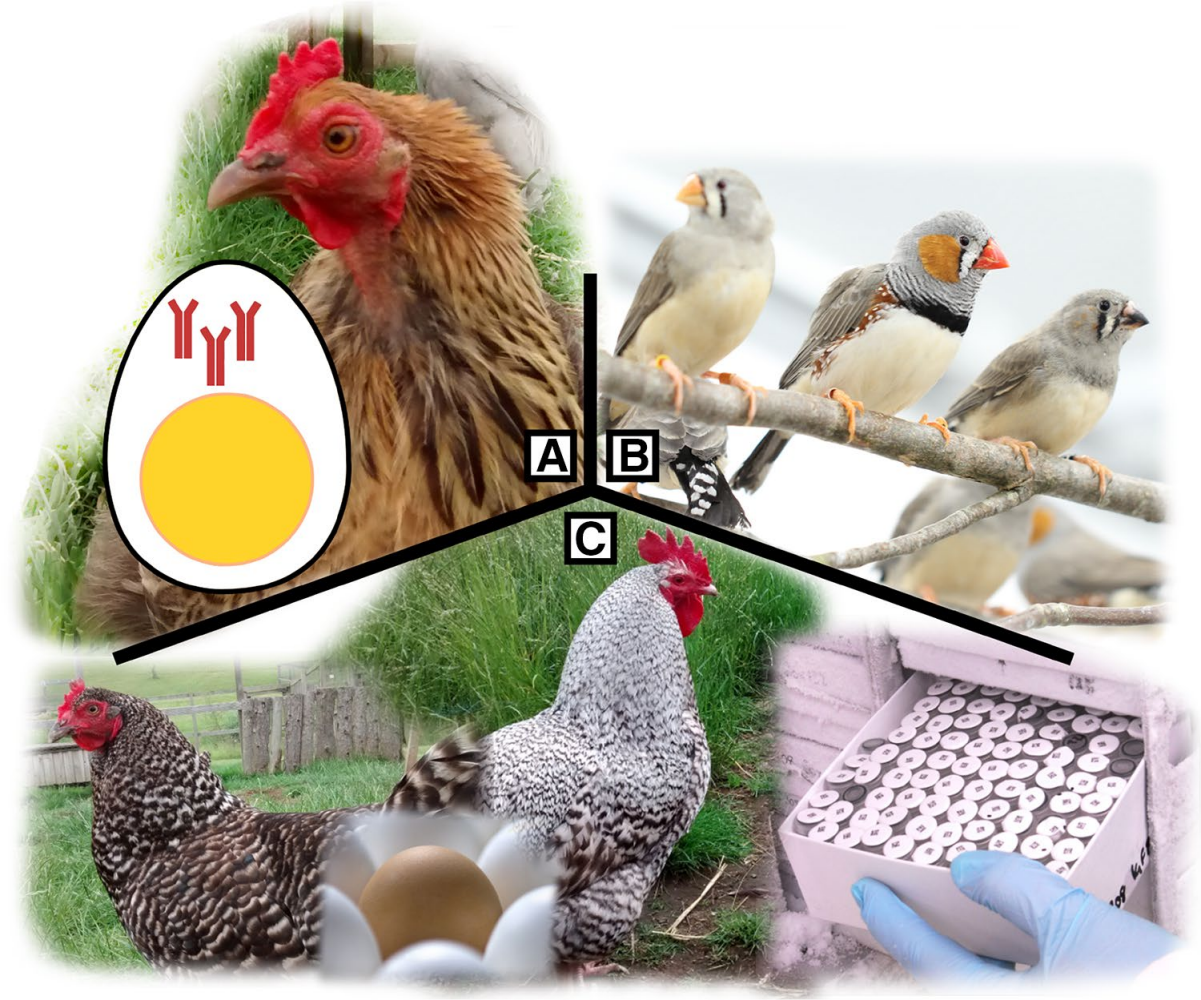
Areas of research and industry which will benefit from advances in avian genome editing technology—**a** production of antibodies and other therapeutic proteins through precise editing of albumen-specific loci, making use of the chicken egg as a pharmaceutical bioreactor. **b** Gene editing in non-poultry avian research models will improve our understanding in areas such as neuroplasticity and senescence. **c** Preservation of valuable genetics across poultry lines, through cryopreservation, and clever use of genetic information to introduce valuable traits into pre-existing lines

Most transgenic chickens bioreactor platforms use regulatory regions from albumen (egg white) expression-specific loci, such as ovalbumin (OVA) locus, with high levels of target protein secreted by cells in the magnum of the oviduct as a result (Zhu et al. 2005; Lillico et al. 2007). Recent reports have demonstrated the production of antimicrobial peptides (Liu et al. 2015a), monoclonal antibodies for breast cancer therapy (Oishi et al. 2011), epitope peptides for pollen (Kawabe et al. 2012) and tissue plasminogen activator for anti-thrombotic therapies (Kaleri et al. 2011; Lee et al. 2013) in egg white. In fact, the first egg-specific pharmaceutical protein, Kanuma, was recently approved for treatment of lysosomal acid lipase deficiency (Sheridan 2016). The flexibility of genome editing will open many avenues for implementing therapeutic protein production in chicken eggs (Fig. 3).

### Models for ageing and behaviour

Bird brains are a useful comparative model for neuroplasticity, with songbirds (e.g. zebra finch and canary), hummingbirds and parrots sharing the human behaviour of vocal learning. Similar to speech acquisition, these birds form long-term memories from birdsong mentors and their song is highly suited for quantitative analysis (Bolhuis and Gahr 2006; Mello 2014). Many birds also show resistance to age-related degenerative processes, despite possessing traits commonly found in short-lived animals such as an elevated body temperature and a rapid metabolic rate. The parallels of age-related disease progression between many bird and mammalian species may reveal novel mechanisms for resistance to senescence with further study (Austad 2011). Transgenic vectors have been used sparingly in song birds (Agate et al. 2009; Velho and Lois 2014; Mak et al. 2015), and have been used to investigate vocal learning (Abe et al. 2015) and neural disorders (Liu et al. 2015b) but this system has proven to be technically difficult. The application of gene editing will conceivably facilitate the use of these bird species as comparative models to current rodent models for learning and age-related brain disorders.

### Poultry production

With 62 billion broilers raised and slaughtered for meat worldwide each year, in addition to 7 billion layers (FAO 2014), chickens represent enormous value to the agricultural industry. Such tremendous numbers are generated by crossbreeding with select standard lines, each line bearing its own commercially desirable traits. Modern breeding methods, i.e. quantitative genetics, have resulted in great advances for traits such as feed conversion and growth. However, there are phenotypic drawbacks to these gains, including reduced fecundity, skeletal defects and other metabolic diseases (Emmerson 1997; Hocking 2010). Furthermore, as a consequence of intensive selection over numerous generations, it is estimated that commercial poultry has lost one-half of its original genetic diversity, such as in chickens, turkey and likely other poultry species (Rathgeber et al. 2013; Aslam et al. 2014; Whyte et al. 2015). Further loss of genetic diversity may be mitigated with cryopreservation strategies, where storage of stem cells or tissue from specialised breeds will allow integration of their genetics into future poultry lines, should their traits become commercially valuable (Liu et al. 2013).

The use of genetic markers to assist selection can provide great benefit to breeding programmes. In cases such as disease resistance and immune function, pedigree stocks are challenged without the need for disease exposure, and aspects such as environmental variance can be easily controlled (Wheeler 2003; Fulton 2004). But genetic selection may not be enough by itself to achieve the value required in traits targeted for future commercial breeding. Genome editing can provide additional benefit, through either production of novel markers or manipulation of the genome to introduce new traits. Recently, chickens with reduced transmission of avian influenza virus were produced by lentiviral transfection of embryos to insert an RNA hairpin molecule into the genome to interfere with viral replication (Lyall et al. 2011). Greater understanding of the pathogenicity of specific diseases could open new avenues for avian disease management, through the application of genome editing.

## Concluding remarks

Editing of the chicken genome is becoming a routine practice in several avian research institutes. With rapid improvements to the reference genome and the cost effectiveness of modern deep sequencing, there are increasing opportunities to target regions of the avian genome for industrial or research applications. Technological improvements will be required for the genetic manipulation of non-chicken avian species to introduce gene edits that persist through the germline. The adaption and application of editing technology for use in other amniote organisms (such as non-avian reptiles), as yet untouched by genome editing, will prove invaluable in the years to come. These advances will be supported by improvements to avian PGC culture, artificial insemination with transfected sperm or the production of suitable surrogate hosts to carry gene-edited PGCs to term.
